# Changes in take-home aerated soft drink purchases in urban India after the implementation of Goods and Services Tax (GST): An interrupted time series analysis

**DOI:** 10.1016/j.ssmph.2021.100794

**Published:** 2021-04-20

**Authors:** Cherry Law, Kerry Ann Brown, Rosemary Green, Nikhil Srinivasapura Venkateshmurthy, Sailesh Mohan, Pauline F.D. Scheelbeek, Bhavani Shankar, Alan D. Dangour, Laura Cornelsen

**Affiliations:** a Department of Public Health, Environments and Society, London School of Hygiene and Tropical Medicine, 15-17 Tavistock Place, WC1H 9SH, London, UK; b (Honorary) College of Medicine and Health, University of Exeter, Exeter, EX1 2LU, UK; c Faculty of Public Health & Policy, London School of Hygiene & Tropical Medicine, 15-17 Tavistock Place, London, WC1H 9SH, UK; d Centre on Climate Change and Planetary Health, London School of Hygiene & Tropical Medicine, Keppel Street, London, WC1E 7HT, UK; e Centre for Chronic Disease Control, C1/52, 2nd Floor, Safdarjung Development Area, New Delhi, 110016, India; f Public Health Foundation of India, Plot 47, Sector 44, Institutional Area Gurugram, 122002, India; g Centre for Chronic Conditions and Injuries (CCCI), Plot 47, Sector 44, NCR, Gurgaon, Haryana, 122002, India; h Institute for Sustainable Food and Department of Geography, University of Sheffield, Winter St, Sheffield, S3 7ND, UK

**Keywords:** Tax, India, Soft drinks, Interrupted time series, Consumer purchase

## Abstract

**Objectives:**

Taxes on sugar-sweetened beverages (SSB) are increasingly being implemented as public health interventions to limit the consumption of sugar and reduce associated health risks. In July 2017, India imposed a new tax rate on aerated (carbonated) drinks as part of the Goods and Services Tax (GST) reform. This study investigates the post-GST changes in the purchase of aerated drinks in urban India.

**Methods:**

An interrupted time series analysis was conducted on state-level monthly take-home purchases of aerated drinks in urban India from January 2013 to June 2018. We assessed changes in the year-on-year growth rate (i.e. percentage change) in aerated drink purchases with controls for contextual variables.

**Results:**

We found no evidence of a reduction in state-level monthly take-home aerated drink purchases in urban India following the implementation of GST. Further analysis showed that the year-on-year growth rate in aerated drink purchases increased slightly (0.1 percentage point per month, 95%CI = 0.018, 0.181) after the implementation of GST; however, this trend was temporary and decreased over time (0.008 percentage point per month, 95%CI = −0.015, −0.001).

**Conclusions:**

In India, a country currently with low aerated drink consumption, the implementation of GST was not associated with a reduction in aerated drink purchase in urban settings. Due to the lack of accurate and sufficiently detailed price data, it is not possible to say whether this finding is driven by prices not changing sufficiently. Furthermore, the impact of GST reform on industry practice (reformulation, marketing) and individual behaviour choices (substitution) is unknown and warrants further investigation to understand how such taxes could be implemented to deliver public health benefits.

## Highlights

• India imposed a new tax rate on aerated drinks as part of the GST reform in 2017.
• We study changes in aerated drink purchases in urban India from 2013 to 2018.
• No evidence for post-GST decrease in state-level monthly take-home drink purchases.
• Need further research to identify the reasons behind the post-GST purchase trend.

## 1 Introduction

Sugar-sweetened beverages (SSBs), such as energy drinks, flavoured juice drinks and carbonated drinks (or aerated drinks as known in India), are recognised as major contributors to sugar consumption and its associated health risks (e.g. dental caries, obesity and diabetes) (World Health Organization, 2017). An increasing number of countries have enacted taxes on SSBs as a strategy to reduce sugar consumption and improve population health (Allcott, Lockwood, & Taubinsky, 2019b; Cawley, Thow, Wen, & Frisvold, 2019). These interventions have generally been considered effective at increasing prices and reducing purchases of SSB, with a 10% increase in sales tax associated with approximately a 10% reduction in SSB purchases (Teng et al., 2019).

As part of an approach to reduce the rising burden of non-communicable chronic diseases among the Indian population, the Food Safety and Standards Authority of India (FSSAI) proposed limiting SSB consumption and introducing additional taxes on sugar-sweetened aerated drinks (FSSAI, 2017). In July 2017, the Indian government implemented the Goods and Services Tax (GST) reform which imposed a 40% tax rate on aerated drinks (The Times of India, 2017a). The GST subsumed the state-level value added tax, national excise duties and several other taxes into a single system. It was levied on the value added at every stage of the supply chain, with tax rates ranging from 0% to 28%. For some luxury and ‘sin’ goods (e.g. tobacco, cigarettes, motor vehicles), an additional tax rate, called ‘compensation cess’, was also imposed to compensate, primarily manufacturing states, for any tax revenue loss due to the change in the system from a production tax towards a consumption tax (Financial Times, 2017; The Times of India, 2017a). Aerated drinks were the only food and beverage good that faced both the highest GST tax rate (i.e. 28%) and a 12% cess, and thus a total tax rate of 40% (Government of India, 2019a). Prior to the GST reform, tax rates varied across states and the exact rate that prevailed in each state is unknown. It is generally accepted, however, that the GST reform increased tax rates on aerated drinks across all states in India (Business Wide India, 2016; The Economic Times, 2016).

No studies have, to date, investigated the impact of the GST reform on aerated drink consumption in India. At the national level, sales of aerated drinks have increased, in recent years, although the growth rate is slower than for other types of drinks. From 2016 to 2019, aerated drinks sales volume in India increased from 5316 million litres to 6515 million litres, a 22.5% increase in four years. During the same period, the total sales volume of all soft drinks increased by 24.8%. Juices, in particular, experienced a rapid growth of 31.9% in sale volume (Euromonitor, 2019). The national average retail price of aerated drinks only increased by 3.7% from INR 59.61/litre in 2016 to INR 61.85/litre in 2018, which was lower than the price rise in juices (10.5%) and the soft drink market as a whole (5.9%).^1^ This indicates that despite the seemingly high tax rate, the pass through of the GST on retail prices of aerated drinks is likely to have been low.

Compared to the taxes on SSBs in other countries, the high tax rate on aerated drinks in India has two key differences. First, although following FSSAI recommendations, the primary aim of this tax was not to improve public health. The introduction of GST is commonly described as a means to simplify the tax system between federal states and increase transparency and efficiency of trade (John, Dauchy, & Goodchild, 2019). Second, per capita consumption of sugary drinks in urban India is relatively low and it is unclear whether SSB taxation policy is effective in countries with low baseline consumption. SSB taxation is seen to have reduced SSB consumption where the baseline consumption levels are high. For example, in Mexico and Chile where 173 L and 179 L of SSBs were sold per person by retailers such as supermarkets and grocery stores in 2017, respectively (Arteaga, FLores, & Luna, 2017; Colchero, Guerrero-López, Molina, & Rivera, 2016; Colchero, Popkin, Rivera, & Ng, 2016; Euromonitor, 2019; Nakamura et al., 2018). In contrast, a recent study in urban India suggested relatively low annual purchases of sugary drinks (aerated drinks, juices, milk-based drinks, squashes and powdered drinks) for consumption at home, which was estimated to be 1.11 L per capita in 2017 (Law et al., 2019). Given this limited per capita consumption of sugary drinks in India, whether a SSB tax remains effective in this context is worthy of investigation.

The aim of this paper is to estimate state-level changes in take-home purchases of aerated drinks in urban India following the introduction of the GST and the compensation cess. This study contributes to the wider literature on SSB taxes in two ways. First, to our best knowledge, it is the first quantitative evaluation study of a SSB tax from the Asian region. Second, as most existing studies on SSB taxes come from countries with relatively high SSB consumption level, this quasi-experimental setup provides an opportunity to understand how a non-health specific tax in a setting with a low per capita consumption may affect purchases.

## 2 Material and methods

### 2.1 Data

This study used a novel state-level dataset on the monthly total volume of aerated drink purchases for consumption at home over the period between January 2013 and June 2018. This includes 12 months post-GST reform data, allowing us to examine the short-term changes in aerated drink purchases. The dataset was constructed from the purchase records of an on-going demographically representative urban Indian household panel, provided by the market insight company, “Kantar – Worldpanel Division, India”. Households were invited to participate in this panel based on their occupational socio-economic status, age of the person responsible for food purchase as well as the state of domicile (Law et al., 2019).^2^ The primary shoppers of the participating households were asked to fill in paper diaries to record all take-home purchases. Purchases made for out-of-home consumption were excluded. The paper diaries covered the volume of purchases but did not collect information on price and monetary expenditure. To ensure that purchases were recorded correctly, interviewers from ‘Kantar – Worldpanel Division, India’ regularly checked the information in the paper diaries against packaging and wrappers collected by households in pre-provided containers. These records included purchases of branded aerated drinks produced by international beverage companies (e.g. Coca Cola and Pepsi) and local companies (e.g. Jayanti, Campa and Appy Fizz) as well as unbranded drinks.

During the data period, 48,490 unique urban households in the panel reported purchases of aerated drinks at least once. Of those who purchased aerated drinks the average purchase was 3.08 L per household per month with standard deviation of 3.98 L. It should be noted that these figures are only broadly indicative as they are not adjusted by survey weight and therefore not demographically representative of all urban India. Furthermore, they are likely to be an overestimate of monthly purchase of aerated drinks per urban household given that we did not have records on households who were in the panel but did not purchase aerated drinks at that time.^3^ Due to these data issues, it would be problematic to conduct the analysis on aerated drink purchases at household level. We therefore aggregated the purchase records to state-level using survey weights.

Data was aggregated to the state rather national level because of differences in pre-GST tax rates on aerated drinks, as well as purchase volumes of sugary drinks between states (Law et al., 2019). While we were unable to identify the exact tax rates imposed by each state for the period 2013–2017 from official sources, the Indian Beverage Association (IBA) provided an overview of variations in tax rates on aerated drinks across states, in their press release in November 2016, which are presented in Table 1 (Business Wide India, 2016). In the majority of states, the total tax rates on aerated drinks ranged from 25.1% to 27.6%. To raise money for farmers affected by the drought, a few states levied a temporary surcharge (i.e. “drought tax”) at that time which increased the total tax rates on aerated drinks to over 30%. While no further information on the exact states under each tax rate were provided by the IBA, they clarified that the highest state tax rate (i.e. 30.25%) was only applicable in Punjab. This suggested that Punjab was the only state that could have possibly reduced its tax rate (i.e. from 42.85% to 40%, including cess) on aerated drinks after the implementation of GST. It should also be noted that there might have been further changes in tax rates on aerated drinks prior to the implementation of GST as some sources cited that the total tax rates in India were 32%–35% in May–June 2017, although no further information at state-level was provided (The Economic Times, 2017; The Times of India, 2017a).

**Table 1:** Pre-GST Tax rates on aerated drinks across states and union territories in India (as of November 2016).

No of states	Central effective tax rate	Approximate state tax rate	Total tax rate
4	12.6%	12.5%	25.1%
4	12.6%	13.5%	26.1%
12	12.6%	14.5%	27.1%
4	12.6%	15%	27.6%
5	12.6%	20%^a^	32.6%
1	12.6%	25%^a^	37.6%
1	12.6%	30.25%^b^	42.85%

a These states increased the tax rates on aerated drinks temporarily to raise revenue to assist farmers affected by the drought.

b This rate was applicable at the first point of sale in the State of Punjab only.

To construct the state-level dataset, we first computed the demographically weighted sum of purchases to estimate total purchases of each state in each month (see Fig. D1 in supplementary materials for graphical presentation). Across all states, the monthly aerated drink purchases were typically higher in the summer months (June and July). The state-level monthly purchase estimates were then pooled to form our panel dataset. In total, our state-level dataset covers total take-home purchases of aerated drinks made by urban households from 14 Indian states and one union territory (Delhi) (listed in Table 2).

**Table 2:** Average monthly purchases of aerated drinks a year before and after GST in urban India (in thousand litre).

	Pre-GST	Post-GST	Difference	95% confidence interval^b^	
	(July 2016–June 2017)	(July 2017–June 2018)			
Delhi	7969	6662	−1,307^c^	(-1811;	−804)
Punjab/Haryana^a^	5424	5250	−174	(-554;	207)
Andhra Pradesh	2610	2941	331^c^	(55;	606)
Uttar Pradesh	2164	2354	190	(-256;	634)
Maharashtra	2109	2255	146	(-51;	343)
Tamil Nadu	2091	1971	−120	(-362;	122)
West Bengal	1334	1099	−235^c^	(-426;	−45)
Karnataka	1119	1045	−74	(-264;	114)
Gujarat	703	671	−32	(-227;	163)
Rajasthan	572	467	−105	(-259;	48)
Orissa	352	436	84^c^	(12;	155)
Bihar	278	322	44	(-19;	107)
Jharkhand	211	177	−34	(-94;	26)
Madhya Pradesh	133	125	−8	(-39;	23)
Kerala	122	73	−49^c^	(-56;	−41)

a These two states are not separated in the data.

b Computed based on standard errors from paired t-tests.

c Statistically significant at 5% level (CI excludes 0).

Apart from aerated drinks, tax rates imposed on other foods and beverages, goods and services were also changed under the GST reform. Depending on their pre-GST tax system, the Indian states might have experienced an increase or a decrease in overall price level of consumer goods after the implementation of GST. We therefore obtained state-wise monthly data on Consumer Price Index (CPI) from the Government of India to account for the overall price effects of the GST (Government of India, 2019b). For Punjab/Haryana, an average CPI among these two states was used.

### 2.2 Empirical strategy

Prior to estimation, we tested the time series of the state-level purchase of aerated drinks to check whether their statistical structures were constant over time (i.e. stationary). The test results are reported in Table C1 in the supplementary materials. There was some evidence of statistical inference problems for the data series for some states. To address this issue as well as the seasonality observed previously, we applied seasonal differencing by computing the year-on-year growth rate, as the percentage change in the state-level purchase of one month relative to the same month in the previous year. Further statistical tests showed some remaining statistical concerns over the data series of Rajasthan, which was therefore dropped in the main analysis to ensure that our results were not subject to estimation bias.

We conducted an interrupted time series (ITS) analysis of year-on-year growth rate of urban aerated drink purchases in 15 Indian states. The monthly state-level data spanned from January 2013 to June 2018, thus providing all combined N = 756 observations. Previous ITS analyses typically model the potential tax impacts on SSB consumption or purchases as a step change that occurred immediately after the tax implementation (Colchero, Guerrero-Lopez, Cummins, & Gasparrini, 2016; Nakamura et al., 2018). However, it is reasonable to expect that there may have been a delay in the effect of GST on aerated drink purchases because of the large overhaul of the whole tax system which could take time for each individual producer and vendor to get used to as well as for consumers to respond to the price changes. In other words, the GST was more likely to have had a gradual impact on purchases over time rather than the immediate level impact seen in other studies (Lopez et al., 2016). We therefore adopted an ITS model that captured linear trend changes over a period (model 1). Specifically, we regressed the year-on-year growth rate on a time variable (i.e. Trend)^4^ and an interaction variable between this time variable and an intervention variable indicating the post-GST period. The coefficient of this interaction variable would capture the average post-GST changes in the year-on-year rates of aerated drink purchases across states.

In addition, we estimated an ITS model that allowed this post-GST change in trend to be non-linear, in other words, to allow that magnitude of the post-GST change in trend to vary over time (model 2). To do so, we included a quadratic term of the time variable (i.e. Trend^2^) and the corresponding interaction term with the GST intervention variable. Model outcomes were derived using ordinary least squares regression controlling for seasonality through month fixed effects.^5^ State-level urban monthly CPI was used to capture price changes in other goods and services. We also included state fixed effects through dummy variables for each state to account for the heterogeneity across states, such as the pre-GST reform tax system, pass-through rates of taxes on consumers, income, population size and food prices. Standard errors were clustered at the state-level. The technical specification can be found in the supplementary materials.

As a robustness check, we performed a sensitivity analysis including observations from Rajasthan. To understand whether our results were driven by one particular state, we re-estimated the models with observations from one state excluded each time. This sensitivity check was particularly important for Punjab as it was the only state, indicated in Table 1, that might have experienced a decline in tax rate on aerated drinks after the GST reform. Additionally, we predicted the fitted values of year-on-year growth rate with estimates from the models and plotted them against the actual values to assess model fit. We then checked if the pooled estimated post-GST changes were robust to the case when observations from states with poor model fit were excluded.

## 3 Results

### 3.1 Main results

**Fig. 1:**
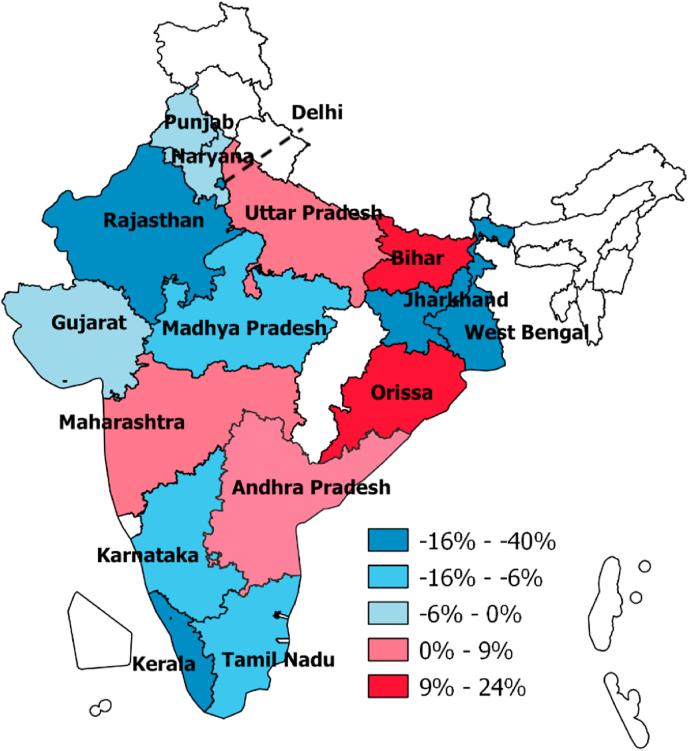
Percentage changes in average monthly purchases of aerated drinks a year before and after GST in urban India * Map represents state lines at the first year of data collection (2013).

Table 2 summarises the average monthly purchases of aerated drinks across states a year before the implementation of GST (June 2016–June 2017) and the year after (July 2017–June 2018) in urban India. For both periods, Delhi had the highest average monthly state-level purchases, followed by Punjab/Haryana and Andhra Pradesh. Kerala was the state with the lowest average monthly purchases of aerated drinks. We also calculated the changes across the two periods by states in Table 2. Out of the 15 states, three saw a significant decline in average monthly purchases of aerated drinks (Delhi −1.31 million litres, West Bengal −0.24 million litres and Kerala −0.05 million litres) whereas two saw an increase (Andhra Pradesh 0.33 million litres and Orissa 0.08 million litres). Fig. 1 illustrates the percentage changes in average monthly purchases of states covered in our dataset, ranging from 24% to −40%. This wide range of percentage changes did not appear to be correlated to the level of monthly purchases at each state. For example, Maharashtra and Tamil Nadu experienced a 7% increase and a 6% decrease in their average monthly purchase of aerated drinks after the implementation of GST respectively although their pre-GST purchases were at a similar level.

To better understand the changes in state-level aerated drink purchases after the implementation of the GST, two ITS models were estimated with controls for underlying purchase trends, state heterogeneity and consumer price level. Table 3 first presents the estimates of model 1. It demonstrates that a slightly negative trend in the year-on-year growth rate of aerated drink purchases ($\beta_{1}$=-0.013, 95% CI: 0.026, −0.001). The estimate of post-GST change in trend ($\beta_{2}$) was 0.019 (95% CI: 0.007, 0.045), suggesting that the GST was not negatively associated with the year-on-year growth rate of aerated drink purchases.

**Table 3:** ITS estimates of post-GST changes in state-level purchases of aerated drinks in urban India.

	Model 1: Linear trend change				Model 2: Non-linear trend change			
	Coefficient	p-value	95% CI		Coefficient	p-value	95% CI	
Trend ($\beta_{1}$)	−0.013	0.006	(-0.026,	−0.001)	−0.016	0.008	(-0.031,	0.0002)
Post-GST change in trend ($\beta_{2}$)	0.019	0.013	(-0.007,	0.045)	0.100	0.042	(0.018,	0.181)
Trend^2^ ($\beta_{3}$)					−0.000	0.003	(-0.006,	0.006)
Post-GST change in Trend^2^ ($\beta_{4}$)					−0.008	0.004	(-0.015,	−0.001)
CPI	0.015	0.012	(-0.008,	0.038)	0.012	0.012	(-0.011,	0.035)
Constant	−1.996	1.554	(-5.042,	1.050)	−1.656	1.961	(-5.500,	2.188)
R-squared		0.095				0.103		
Observations		756				756		

Note: The dependent variable is the year-on-year percentage change in state-level purchases of aerated drinks. Both models are estimated with Ordinary Least Squares and include month and state fixed effects to account for state heterogeneity and seasonality. Standard errors are clustered at state level.

In model 2, we assumed that the changes in the post-GST trend of year-on-year growth rate were not constant over time. The underlying trend of aerated drink purchases remained negative and was of similar statistical significance (i.e. $\beta_{1}$ = -0.015, 95% CI: 0.031, −0.0002). This trend did not seem to change over time as the quadratic trend term ($\beta_{3}$) was close to zero (−0.0001, 95%CI: 0.006, 0.006). The GST intervention was found to be associated with an increase in the trend of the year-on-year growth rate of aerated drink purchases although the magnitude of this increase was small ( $\beta_{2}$ = 0.1, 95%CI: 0.018, 0.1823). This positive change in post-GST trend also appeared to decrease over time as the corresponding estimate on Trend^2^ ($\beta_{4}$) was negative (−0.008, 95%CI: 0.011, −0.001). In both models, we did not find evidence for a negative post-GST change on the year-on-year growth rate of aerated drink purchases.

### 3.2 Sensitivity analysis

For brevity, we focus the sensitivity checks of model 2 which displayed evidence for a non-linear positive post-GST change in the trend of the year-on-year percentage change of aerated drink purchases.^6^ With the inclusion of Rajasthan, the GST reform was found to be associated with a slightly larger positive change on the trend of year-on-year growth rate of aerated drink purchases ($\beta_{2}$=0.15, 95% CI:0.025, 0.275) that decreased at a rate of 0.01 per month ($\beta_{4}$=-0.01, 95% CI: 0.018, −0.002). We also tested the sensitivity of our results to the exclusion of observations by each individual state. We continued to find a positive non-linear post-GST change in trend although the magnitude of this change differed slightly across estimations. In particular, the estimates remained qualitatively the same when observations from Punjab/Haryana were excluded, suggesting that our results were unlikely to be driven by the potential decline in tax rate on aerated drinks in Punjab.

To assess whether model 2 sufficiently captured the information in our state-level purchase data, we plotted the fitted values across states against their actual values in Fig. D2 in the supplementary materials. Visual inspection indicated large gaps between the actual and the fitted values for West Bengal, Orissa, Bihar and Jharkhand reflecting that the model may not have adequately explained the year-over-year percentage change of aerated drink purchases for these states. Consequently, we excluded the observations of these states as well as that of Rajasthan and estimated model 2. The non-linear post-GST change in trend were then found to be not statistically different from zero ($\beta_{2}$=0.077, 95% CI: 0.041, 0.183; $\beta_{4}$ = -0.006, 95% CI: 0.014, 0.004). There remained no evidence in support a decline in the year-on-year growth rate of aerated drink purchases in urban India.^7^

### 3.3 Stratified analysis

We divided our samples into higher and lower income states based on their percentage of urban population living under the poverty line in 2011/12 (Law et al., 2019) and estimated the ITS models on these two samples separately. As taxes on SSBs are typically regressive, aerated drink purchases in states with lower income may have been more sensitive to the implementation of GST. We reported the post-GST estimates of models 1 and 2 in Table 4, which should be interpreted with caution due to the small number of observations in each sample (N = 378).^8^ While the signs of the non-linear post-GST change in trend for both sub-samples were consistent with our main findings, this change was only statistically significant at 5% level for the lower income urban states ($\beta_{2}$=0.170, 95% CI:0.026, 0.316; $\beta_{4}$ = -0.014, 95% CI: 0.026, −0.001).

**Table 4:** Stratified analysis: ITS estimates of post-GST changes in state-level purchases of aerated drinks in urban India.

	Model 1: Linear trend change				Model 2: Non-linear trend change			
	Coefficient	p-value	95% CI		Coefficient	p-value	95% CI	
Panel A: higher income urban states* (N = 378)								
Post-GST change in trend ($\beta_{2}$)	−0.001	0.960	(-0.034,	0.033)	0.037	0.521	(-0.096,	0.171)
Post-GST change in trend^2^ ($\beta_{4}$)					−0.002	0.593	(-0.012,	0.008)
***Panel B: Lower income urban states^ (N = 378)***								
Post-GST change in trend ($\beta_{2}$)	0.035	0.170	(-0.020,	0.092)	0.170	0.028	(0.026,	0.316)
Post-GST change in trend^2^ ($\beta_{4}$)					−0.014	0.042	(-0.026,	−0.001)

Note: *states with percentage of urban population above poverty line in 2011/12 < 10.5%, including Kerala, Delhi, Tamil Nadu, Maharashtra, Andhra Pradesh, Gujarat, Punjab/Haryana. ^states with percentage of urban population above poverty line in 2011/12 > 10.5%, including West Bengal, Karnataka, Orissa, Madhya Pradesh, Jharkhand, Uttar Pradesh, Bihar. The dependent variable is the year-on-year growth rate in state-level purchases of aerated drinks. All models include Trend, Trend^2^, CPI, month and state fixed effects and are estimated using Ordinary Least Squares. Standard errors are clustered at state level. Full model results can be found in Tables E1 -E2 in supplementary materials.

## 4 Discussion

Our findings contribute to the ongoing research on the effectiveness of taxes on SSBs. In contrast to other countries like Mexico and Chile, India presents an unusual context where a tax has been implemented on soft drinks while the per capita consumption is still relatively low. While typical assessments of such taxes start with analysing changes in prices due to the tax, there is a lack of data at state or more disaggregated level on prices of taxed drinks and other products in India. In this study we thus focused on assessing changes in purchase volumes.

Our analysis showed that the implementation of the GST was not associated with a negative change in the year-on-year growth rate of state-level monthly take-home aerated drink purchases in urban India. Our estimates indicated that the year-on-year growth rate of aerated drink purchase volumes increased slightly (0.1 percentage point per month) after the implementation of GST but this trend disappeared over time (0.008 percentage point per month). The sensitivity analysis, which excluded four states, where the fit of the model appeared poorer, weakened these effect sizes: they were no longer significant at conventional statistical significance levels. Owing to lack of data on beverage prices, we cannot analyse to what extent the post-GST changes in prices would explain these findings. Nonetheless, this study is an important first step in assessing changes in purchases of aerated drinks after the GST and compensation cess were implemented in India, as opposed to modelling studies that have predicted the future consumption of products following the implementation of a SSB tax or the GST reform (Basu et al., 2014; John et al., 2019).

Our study provides the first piece of evidence on the potential impact of SSB taxes from an Asian region. While other Asian countries such as the Philippines and Thailand have implemented SSB taxes, these interventions have not, to date, been evaluated. Our findings of a temporary positive post-GST trend differ from previous studies conducted in LMICs, where decreases of 6.1% (Mexico) and 21.6% (Chile) were reported for SSB purchases following the implementation of a SSB tax. Our findings are, however, consistent with the argument that SSB taxation may not always have a significant impact on consumption patterns when the baseline tax rate is already considered high (Jou & Techakehakij, 2012). In India, aerated drinks faced a total tax rate of 40% and yet, the actual increment across states was much lower, 7–15% (when compared to combined central and state tax rates prior to the GST reform). At the same time, the lack of association is not unprecedented. For example, a recent study found no negative association between a SSB tax and beverage purchases at 12 months post-tax time points by comparing purchases made by residents in two US cities, one with and one without the SSB tax (Lawman et al., 2020). A systematic review of real-world SSB tax evaluation in 2019 also reported a minority of studies that evaluated SSB taxes in the US and found no evidence of a negative impact on SSB consumption (Teng et al., 2019).

While sufficiently detailed price data is lacking in India, market reports from Euromonitor International suggest, at the national level, a limited increase in retail prices of aerated drinks. Their figures show the total sales volume of aerated drinks increased by 8% per year in 2018 and 2019, while the total sales values (at retail selling prices) rose by 10% per year.^9^ The slightly faster growth rate of sales value than volume suggests that retail prices of aerated drinks increased but likely at a low rate. This suggests that only a small amount of the price increment from the GST reform was passed to consumers, limiting the potential GST effect in reducing purchases.

In addition to tax pass-through rates, the effectiveness of SSB taxes is also subject to price elasticity of demand. If its demand were highly price elastic, the limited increase in retail prices would still have a negative impact on aerated drink purchases. However, if the demand were inelastic, little change in purchases would be expected if prices rose by only a small amount. One key determinant of price elasticity of demand is the proportion of income spent on these drinks. Wealthier households tend to be less sensitive to price changes in aerated drinks as they only spend a small percentage of their income on buying these drinks (Muhammad, Meade, Marquardt, & Mozaffarian, 2019). If consumption of aerated drinks is largely concentrated among wealthier households in urban India, then it is possible that the findings of this study are reflecting inelastic demand. A careful examination of the pass-through rates and price elasticity of demand is therefore crucial when designing fiscal measures to discourage SSB consumption.

Another reason that might explain the limited post-GST negative changes seen in the current study could be the lack of public engagement or awareness with the health effects of consuming SSBs. The additional tax on aerated drinks was part of a major tax reform in India, which affected a variety of industries and products. It was not specifically introduced as a health-related tax, and whilst some media highlighted the high rate of GST on aerated drinks, no clear rationale on the health impacts was provided (The Economic Times, 2017; The Times of India, 2017b). This meant that there was limited public debate on the likely health effects of aerated drinks, or any “signalling effect” to encourage people to be more conscious about their beverage choices (Hilton et al., 2019). In contrast, the debate around the SSB tax attracted a considerable amount of media attention in Mexico, which increased the public visibility of the health messaging about SSBs, and might explain why the SSB tax was considered more effective in this setting (Donaldson, 2015; Álvarez-Sánchez et al., 2018).

The temporary rise in growth rate of purchases observed could have been due to industry reactions towards the GST reform. Aerated drink companies introduced new variants and focused on innovative marketing to bring consumers back to these drinks (Euromonitor, 2019). Local aerated drink producers were able to avoid the high tax rate by adding 10% fruit juice into carbonated drinks, as the 40% rate was levied on sugary fizzy drinks with no fruit content and fruit-based beverages fell under the 12% GST rate (The Times of India, 2018b). This means some juice carbonates could have been cheaper under the GST regime (Euromonitor, 2019). Indeed, as discussed earlier, the retail prices of aerated drinks increased at a much slower rate than juices and the soft drink market as a whole. By changing drink recipes, the industry could minimise the actual price increment of aerated drinks, which is the primary mechanism to reduce take-home purchases. Reports indicated Coca-Cola India and PepsiCo India witnessed a recovery of volume growth in 2018, driven by their strong marketing campaigns (Euromonitor, 2019). These industry responses might have counteracted any simultaneous downward pressure on purchases from the GST reform. These potential unintended consequences imply that the increased tax rate on aerated drinks might not have lowered sugar consumption among the Indian population. This limits the effectiveness of the SSB tax in achieving FSSAI's objective to mitigate the rising burden of non-communicable chronic diseases in India.

As with any studies, this paper has limitations. Our analysis relied on the pre- and post-GST introduction time dimension to identify the average post-GST changes across all states, as we did not find sufficiently detailed state-level information on the pre-GST tax levels of aerated drinks for the study period. Future research could identify state-specific effects of the GST through in-depth investigation into the changes in tax system as well as prices of aerated drinks in particular states, such as Delhi which had the highest volume of aerated drink purchases at state-level.

Second, we were not able to use a control group in our analyses, which would have added robustness to the findings. This was not possible because the GST reform was a national policy and no states would be exempt to act as control groups. It was also a fundamental reform in the indirect tax system, which subsumed services tax, state-level value added tax and some other taxes. It was likely to impact most industries and, therefore, difficult to identify a product not affected by the GST, which could act as a control group. To minimise the bias from concurrent events, we included the state-level CPI in our models. This allowed us to control for the changes in the state-level general prices and ensure our estimates reflected the impact of any potential GST-induced changes in the relative price of aerated drinks on the purchases.

Third, our dataset did not cover purchases in rural India. In 2011–12, urban households consumed 82 ml cold beverages and 53 ml fruit juices per capita per 30 days, which were much higher than rural households (38 ml and 10 ml respectively) (NSSO, 2014). While urban India remains the dominant market for soft drinks, rural sales have been growing in the past few years (The Times of India, 2018a). The implementation of GST could have more noticeable impacts on aerated drink purchases in rural India where households tend to be poorer than their urban counterparts and less likely to have a strong habit of consuming aerated drinks. Therefore, rural households could be more sensitive to price changes caused by the GST reform and more likely to adjust their purchase patterns. This potential negative impact might, however, be counteracted by the industry's plan to increase direct distribution and drive deeper penetration into rural markets (The Times of India, 2018a).

Fourth, our data consists of take-home purchases of aerated drinks. This excludes data on on-the-go or food service purchases (e.g., street vendors or restaurants). We acknowledge that restaurant and street vendor purchases could represent approximately 40% of the total aerated drink purchases in India by value; however, it is unclear whether including these data would have changed the results of this study as take-home purchases continue to represent the majority of aerated drink consumption, particularly when measured by volume (Euromonitor, 2019). Furthermore, this is a general caveat of most evaluation studies of SSB taxes as detailed data on purchases made for consumption out-of-home is lacking even in high-income countries (Allcott, Lockwood, & Taubinsky, 2019a; Pell et al., 2020).

Lastly, as we did not have detailed data on purchases of caffeinated beverages, water or natural juices, we were unable to identify any substitution away from aerated drinks. In July 2017, the GST rate for natural juices was set at 12% while bottled water was taxed at 18%. After two years of implementation the GST rate of caffeinated beverages was raised from 18% to 40% (28% GST rate + 12% cess), the same rate as aerated drinks (The Economic Times, 2019). It is particularly important to understand the impact of GST on these beverages as the role of aerated drinks has been diminishing – aerated drinks accounted for 66% of the soft drink market in 2004 and this reduced to 26% in 2018 in India. In the contrary, the share of bottled water sales in the Indian soft drink market has grown from 25% in 2004 to 64% in 2018 (Euromonitor, 2019).

## 5 Conclusion

In this study, we employed an interrupted time series design to examine how state-level monthly take-home purchases of aerated drinks, in urban India, changed in the first year after the implementation of GST. We found no evidence of a decline in state-level aerated drink purchases following the GST reform. There are several reasons why no negative post-GST changes in purchases were detected including the high pre-GST tax rates, minimal changes in prices as industries avoided the high tax rate by changing recipes, as well as the limited effects on public awareness as the GST was not a direct health-related tax. Detailed data on state-level pre-GST tax rates as well as market prices of aerated drinks are needed to identify the relative importance of these factors. The limitations of our study reflect the complexity in evaluating the effectiveness of SSB taxes in countries undergoing significant economic and social changes and the need for detailed price and purchase data of aerated drinks and other soft drinks for both take-home and out-of-home consumption to isolate the influence from these changes.

While the findings in this study should not be viewed as conclusive evidence of the effect of the GST on aerated drink consumption in urban India, we shed light on the possibility that the GST in India, that currently has a low consumption of SSBs, has not had the same effect as the SSB taxes implemented in other countries. It is unclear whether this tax in its current form is a sufficient preventative measure to benefit public health in the long-term future.

## Financial Support

This study forms part of the Sustainable and Healthy Food Systems (SHEFS) programme supported by the Wellcome Trust's Our Planet, Our Health programme [grant number: 205200/Z/16/Z]. LC is funded via UK Medical Research Council Fellowship MR/P021999/1. Funding bodies had no role in the data collection, analysis or interpretation, and no role in the study design or in writing the manuscript.

## Authorship

CL and LC conceptualised the study and were responsible for the study design and development of methods. ADD prepared the grant application along with SM, BS, PSDS, RG and LC. CL conducted the literature review for background as well as the data analysis. CL, LC, KAB, RG and ADD drafted the manuscript. NSV, SM, PFDS and BS contributed substantially to data interpretation and provided critical comments on the manuscript.

## Author statement

CL and LC conceptualised the study and were responsible for the study design and development of methods. ADD prepared the grant application along with SM, BS, PSDS, RG and LC. CL conducted the literature review for background as well as the data analysis. CL, LC, KAB, RG and ADD drafted the manuscript. NSV, SM, PFDS and BS contributed substantially to data interpretation and provided critical comments on the manuscript.

## Ethical statement

This study does not involve human subject as it uses secondary anonymous data. It is the authors’ own original work and has not been previously published elsewhere.

## Declaration of competing interest

None.

## Footnotes

1 Figures on sales volume of all soft drinks, juices and aerated drinks from 2013 to 2019 can be found in the supplementary materials.

2 Previous study compared the estimates of per capita annual purchase in urban India from the NSSO data in 2011–12 with the data from “Kantar – Worldpanel Division, India” in 2013 over comparable foods and found that the discrepancy between estimates is small (Law et al., 2019). These comparitions support the credibility of the dataset used in this study.

3 For example, let us say that the last reported purchase of aerated drinks of a household was in January 2018. If this household left the panel, their purchases onward would be coded as missing. However, if they remained on the panel but did not purchase any more aerated drinks in 2018, their purchases onward would be coded as zero. Since we were unable to tell when the households entered and left the panel, it was not possible to code missing or zero household purchase accurately and hence to analyse the data at household level.

4 For easier interpretation, the time variable is constructed with June 2017 as the reference period.

5 All data analyse were performed with Stata 15.

6 Detailed results of the sensitivity analysis can be found in supplementary materials.

7 In supplementary materials, we present a robustness check with the inclusion of annual state-wise net domestic product to account for potential income effects. The results are qualitatively consistent with our main findings.

8 Full results are available in the supplementary materials.

9 See supplementary materials for the precise figures on the sales values and volume.
