# Supplementary material for: Changes in take-home aerated soft drink purchases in urban India after the implementation of Goods and Services Tax (GST): An interrupted time series analysis
Source: SSM Popul Health. 2021 Apr 20;14:100794. doi: 10.1016/j.ssmph.2021.100794 (PMC8102159; doi:10.1016/j.ssmph.2021.100794)
Supplement: Multimedia component 1 [file mmc1.docx]

**Changes in take-home aerated soft drink purchases in urban India after the implementation of Goods and Services Tax (GST): an Interrupted Time Series analysis**

**Supplementary materials**

1. **Sales of soft drinks, aerated drinks and juices in India from 2013 to 2019**

|  | **2013** | **2014** | | **2015** | | **2016** | | **2017** | | | **2018** | | | **2019** | | |  |  |
| --- | --- | --- | --- | --- | --- | --- | --- | --- | --- | --- | --- | --- | --- | --- | --- | --- | --- | --- |
| **Soft drinks*** | | | | | | | | | | | | | | | | |  |  |
| Volume (million L) | 5658 | 6228 | (10%) | 6733 | (8%) | 7198 | (7%) | | 7605 | (6%) | | 8268 | (9%) | | 8986 | (9%) | |  |
| RSP Value (INR billion) | 418 | 489 | (17%) | 556 | (14%) | 625 | (12%) | | 700 | (12%) | | 806 | (15%) | | 928 | (15%) | |  |
| Average RSP (INR/L)^ | 59.19 | 61.40 | (4%) | 63.66 | (4%) | 65.48 | (3%) | | 67.30 | (3%) | | 69.40 | (3%) | | 71.43 | (3%) | |  |
| **Aerated drinks (i.e. Carbonates)** | | | | | | | | | | | | | | | | | | |
| Volume (million L) | 4302 | 4667 | (8%) | 4990 | (7%) | 5316 | (7%) | | 5586 | (5%) | | 6029 | (8%) | | 6515 | (8%) | |  |
| RSP Value (INR billion) | 237 | 266 | (12%) | 292 | (10%) | 317 | (8%) | | 340 | (7%) | | 373 | (10%) | | 410 | (10%) | |  |
| Average RSP (INR/L)^ | 55.18 | 57.02 | (3%) | 58.60 | (3%) | 59.61 | (2%) | | 60.82 | (2%) | | 61.85 | (2%) | | 62.93 | (2%) | |  |
| **Juices** | | | | | | | | | | | | | | | | | | |
| Volume (million L) | 1276 | 1473 | (15%) | 1647 | (12%) | 1779 | (8%) | | 1911 | (7%) | | 2122 | (11%) | | 2347 | (11%) | |  |
| RSP Value (INR billion) | 82 | 98 | (20%) | 116 | (18%) | 132 | (14%) | | 148 | (12%) | | 174 | (17%) | | 203 | (17%) | |  |
| Average RSP (INR/L)^ | 64.10 | 66.74 | (4%) | 70.26 | (5%) | 74.36 | (6%) | | 77.51 | (4%) | | 82.20 | (6%) | | 86.55 | (5%) | |  |

Note: RSP= Retail selling price. *Soft drinks included carbonates, concentrates, juices, energy drinks, sport drinks and ready-to-drink tea. ^Average RSP is calculated by dividing sales volume in litre with total sale value in RSP. Both figures are obtained from Euromonitor. Figures in parentheses give the percentage change from previous year.

1. **Interrupted time series model**

Model 1: Linear trend change

$${YOY}_{st}=\alpha+ \beta_{1}Trend+ \boldsymbol{\beta}_{\boldsymbol{2}}\boldsymbol{GST}_{\boldsymbol{t}}\boldsymbol{*Trend}+\beta_{c}{CPI}_{st}+\beta_{s}S+\beta_{m}m+\varepsilon_{st}$$

${YOY}_{st}$ is the year-on-year growth rate of aerated drink purchases ($CD$) for state $s$ at month $t$ relative to $t-12$, in other words, ${YOY}_{st}=$ (${CD}_{s,t}-{CD}_{s,t-12})/{CD}_{s,t-12}$. $Trend$ is the time period variable in which it is set to equal 0 in June 2017, the month before the implementation of GST. $Trend$ranges from -53 to 0 before the GST reform and from 1 to 12 after the reform. ${GST}_{t}$ is the intervention variable, which takes the value of 1 from July 2017 when the GST came into effect, and 0 otherwise. ${GST}_{t}$ is interacted with the time period ($Trend$) and $\beta_{2}$ shows the linear post-GST change in purchase trend. This model assumes that the changes in aerated purchases would be gradual in the gradient (slope) of the growth rate. ${CPI}_{st}$ is the CPI of in state $s$ at month $t$. $S$ is a vector of state fixed effects to account for the time-invariant heterogeneity in growth rate of aerated drink purchases across states. As a further control for seasonality, we included a group of month dummies, $m.$Lastly, $\varepsilon_{st}$ represents the error term.

Model 2: Non-linear trend change

$${YOY}_{st}=\alpha+ \beta_{1}Trend+ \boldsymbol{\beta}_{\boldsymbol{2}}\boldsymbol{GST}_{\boldsymbol{t}}\boldsymbol{*Trend}+ \beta_{3}{Trend}^{2}+ \boldsymbol{\beta}_{\boldsymbol{4}}\boldsymbol{GST}_{\boldsymbol{t}}\boldsymbol{*}\boldsymbol{Trend}^{\boldsymbol{2}}$$

$$+\beta_{c}{CPI}_{st}+\beta_{s}S+\beta_{m}m+\varepsilon_{st}$$

${Trend}^{2}$ is a quadratic term describing the time period. Building on model 1, this model does not only assume a gradual post-GST change in year-on-year growth rate of aerated drink purchases, but also allows the magnitude of this change in trend to vary over time, which is captured by $\beta_{5}$, the coefficient of the interaction term between ${GST}_{t}$ and ${Trend}^{2}$. It also includes the state and month fixed effects as well as CPI as control variables.

1. **Additional tables**

Table C1 Augmented Dickey-Fuller Unit root test results

|  | Level^ | |  | Year-on-year growth rate* | |
| --- | --- | --- | --- | --- | --- |
|  | Test Statistics | P-value |  | Test Statistics | P-value |
| Delhi | -2.797 | 0.059 |  | -4.630 | 0.000 |
| Jharkhand | -3.339 | 0.013 |  | -3.713 | 0.004 |
| Andhra Pradesh | -3.923 | 0.002 |  | -3.727 | 0.004 |
| Maharashtra | -4.730 | 0.000 |  | -5.707 | 0.000 |
| Punjab/Haryana | -2.130 | 0.233 |  | -4.723 | 0.000 |
| West Bengal | -4.009 | 0.001 |  | -3.694 | 0.004 |
| Gujarat | -6.189 | 0.000 |  | -6.352 | 0.000 |
| Karnataka | -3.216 | 0.019 |  | -4.416 | 0.000 |
| Kerala | -3.587 | 0.006 |  | -3.523 | 0.007 |
| Rajasthan | -2.895 | 0.046 |  | -2.371 | 0.150 |
| Orissa | -3.419 | 0.010 |  | -6.117 | 0.000 |
| Madhya Pradesh | -3.793 | 0.003 |  | -5.638 | 0.000 |
| Uttar Pradesh | -2.587 | 0.096 |  | -4.922 | 0.000 |
| Tamil Nadu | -3.052 | 0.030 |  | -3.235 | 0.018 |
| Bihar | -2.610 | 0.091 |  | -8.592 | 0.000 |

Note:^5% critical value:-2.918 *5% critical value:-2.928

Table C2 Sensitivity check of the linear trend change model (model 1)

| **Dropped state(s)** | **Trend** | **Post-GST change in Trend** | **CPI** | **Constant** |  |
| --- | --- | --- | --- | --- | --- |
|  | $\beta_{1}$ | $\beta_{2}$ | $\beta_{c}$ | $\alpha$ |  |
| **Panel A: All states (N= 810)** | | | | | |
| None | -0.011 | 0.021 | 0.006 | -0.943 |  |
|  | (0.007) | (0.012) | (0.014) | (1.792) |  |
| **Panel B: Individual state excluded (N= 702)** | | | | | |
| Delhi | -0.012 | 0.023 | 0.010 | -1.180 |  |
|  | (0.007) | (0.014) | (0.012) | (1.541) |  |
| Jharkhand | -0.012 | 0.020 | 0.013 | -1.699 |  |
|  | (0.007) | (0.014) | (0.013) | (1.746) |  |
| Andhra Pradesh | -0.013 | 0.017 | 0.013 | -1.840 |  |
|  | (0.007) | (0.014) | (0.013) | (1.661) |  |
| Maharashtra | -0.015 | 0.019 | 0.018 | -2.395 |  |
|  | (0.006) | (0.014) | (0.012) | (1.531) |  |
| Punjab/Haryana | -0.017 | 0.023 | 0.020 | -2.740 |  |
|  | (0.006) | (0.013) | (0.011) | (1.375) |  |
| West Bengal | -0.014 | 0.022 | 0.016 | -2.121 |  |
|  | (0.007) | (0.014) | (0.013) | (1.733) |  |
| Gujarat | -0.013 | 0.020 | 0.013 | -1.803 |  |
|  | (0.007) | (0.014) | (0.013) | (1.714) |  |
| Karnataka | -0.013 | 0.020 | 0.012 | -1.679 |  |
|  | (0.006) | (0.014) | (0.012) | (1.605) |  |
| Kerala | -0.015 | 0.023 | 0.020 | -2.662 |  |
|  | (0.006) | (0.014) | (0.012) | (1.500) |  |
| Orissa | -0.011 | 0.011 | 0.013 | -1.750 |  |
|  | (0.007) | (0.012) | (0.013) | (1.726) |  |
| Madhya Pradesh | -0.014 | 0.016 | 0.014 | -1.988 |  |
|  | (0.007) | (0.014) | (0.012) | (1.604) |  |
| Uttar Pradesh | -0.014 | 0.019 | 0.016 | -2.164 |  |
|  | (0.007) | (0.014) | (0.013) | (1.651) |  |
| Tamil Nadu | -0.014 | 0.016 | 0.019 | -2.535 |  |
|  | (0.007) | (0.014) | (0.012) | (1.584) |  |
| Bihar | -0.009 | 0.012 | 0.007 | -1.047 |  |
|  | (0.006) | (0.012) | (0.011) | (1.485) |  |
| **Panel C: Multiple states excluded (N= 540)** | | | | | |
| West Bengal, Orissa, Bihar and Jharkhand | -0.001 | 0.007 | -0.006 | 0.673 |  |
|  | (0.006) | (0.011) | (0.013) | (1.748) |  |

Note: Robust standard errors clustered at state level are given in parentheses. All models are estimated with month fixed effects and state fixed effects. In panel B, the GST effects were estimated repeated with a different state dropped each time while observations from Rajasthan were excluded.

Table C3 Sensitivity check of the non-linear trend change model (model 2)

| **Dropped state(s)** | **Trend** | **Post-GST change in Trend** | **Trend^2^** | **Post-GST change in Trend^2^** | **CPI** | **Constant** |
| --- | --- | --- | --- | --- | --- | --- |
|  | $\beta_{1}$ | $\beta_{2}$ | $\beta_{3}$ | $\beta_{4}$ | $\beta_{c}$ | $\alpha$ |
| **Panel A: All states (N= 810)** | | | | | | |
| None | -0.026 | 0.150 | -0.000 | -0.010 | -0.005 | 0.436 |
|  | (0.013) | (0.064) | (0.001) | (0.004) | (0.022) | (2.771) |
| **Panel B: Individual state excluded (N= 702)** | | | | | | |
| Delhi | -0.0155 | 0.1206 | -0.0001 | -0.0092 | 0.0061 | -0.7901 |
|  | (0.0084) | (0.0398) | (0.0003) | (0.0035) | (0.0171) | (2.1485) |
| Jharkhand | -0.0106 | 0.0926 | 0.0001 | -0.0078 | 0.0128 | -1.7653 |
|  | (0.0064) | (0.0438) | (0.0003) | (0.0038) | (0.0181) | (2.3348) |
| Andhra Pradesh | -0.0153 | 0.0949 | -0.0001 | -0.0073 | 0.0100 | -1.4464 |
|  | (0.0089) | (0.0455) | (0.0003) | (0.0039) | (0.0155) | (1.9852) |
| Maharashtra | -0.0171 | 0.0981 | -0.0000 | -0.0075 | 0.0148 | -2.0883 |
|  | (0.0083) | (0.0452) | (0.0003) | (0.0039) | (0.0155) | (1.9849) |
| Punjab/Haryana | -0.0182 | 0.1167 | -0.0000 | -0.0091 | 0.0174 | -2.4542 |
|  | (0.0079) | (0.0413) | (0.0003) | (0.0036) | (0.0148) | (1.8821) |
| West Bengal | -0.0119 | 0.0952 | 0.0001 | -0.0081 | 0.0160 | -2.2322 |
|  | (0.0074) | (0.0441) | (0.0003) | (0.0038) | (0.0169) | (2.1790) |
| Gujarat | -0.0150 | 0.1099 | -0.0000 | -0.0085 | 0.0094 | -1.3862 |
|  | (0.0087) | (0.0445) | (0.0003) | (0.0038) | (0.0166) | (2.1364) |
| Karnataka | -0.0159 | 0.1042 | -0.0001 | -0.0077 | 0.0078 | -1.1704 |
|  | (0.0087) | (0.0463) | (0.0003) | (0.0039) | (0.0150) | (1.9275) |
| Kerala | -0.0156 | 0.1052 | 0.0000 | -0.0083 | 0.0177 | -2.4627 |
|  | (0.0085) | (0.0445) | (0.0003) | (0.0038) | (0.0152) | (1.9444) |
| Orissa | -0.0188 | 0.1058 | -0.0002 | -0.0076 | 0.0059 | -0.9573 |
|  | (0.0083) | (0.0447) | (0.0002) | (0.0039) | (0.0155) | (2.0042) |
| Madhya Pradesh | -0.0164 | 0.0803 | -0.0001 | -0.0058 | 0.0115 | -1.6559 |
|  | (0.0086) | (0.0396) | (0.0003) | (0.0032) | (0.0154) | (1.9843) |
| Uttar Pradesh | -0.0165 | 0.1067 | -0.0001 | -0.0082 | 0.0122 | -1.7441 |
|  | (0.0085) | (0.0445) | (0.0003) | (0.0038) | (0.0163) | (2.0930) |
| Tamil Nadu | -0.0163 | 0.0922 | -0.0000 | -0.0073 | 0.0162 | -2.2455 |
|  | (0.0086) | (0.0440) | (0.0003) | (0.0039) | (0.0154) | (1.9859) |
| Bihar | -0.0134 | 0.0811 | -0.0001 | -0.0059 | 0.0025 | -0.5121 |
|  | (0.0086) | (0.0400) | (0.0003) | (0.0033) | (0.0143) | (1.8452) |
| **Panel C: Multiple states excluded (N= 540)** | | | | | | |
| West Bengal, Orissa, Bihar and Jharkhand | -0.004 | 0.071 | -0.000 | -0.006 | -0.009 | 0.987 |
|  | (0.005) | (0.050) | (0.000) | (0.004) | (0.016) | (2.089) |

Note: Robust standard errors clustered at state level are given in parentheses. All models are estimated with month fixed effects and state fixed effects. In panel B, the GST effects were estimated repeated with a different state dropped each time while observations from Rajasthan were excluded.

Table C4: ITS estimates of post-GST changes with state-wise net domestic product (NDP)^ (N=756)

|  | **Model 1: Linear trend change** | | | |  | | **Model 2: Non-linear trend change** | | | | |  |
| --- | --- | --- | --- | --- | --- | --- | --- | --- | --- | --- | --- | --- |
|  | Coefficient | p-value | 95% CI | | |  | | Coefficient | p-value | 95% CI | | |
| Trend ($\beta_{1}$) | -0.011 | 0.316 | (-0.034, | 0.019) | |  | | -0.012 | 0.143 | (-0.028, | 0.004) | |
| Post-GST change in Trend ($\beta_{2}$) | 0.019 | 0.164 | (-0.088, | 0.046) | |  | | 0.010 | 0.031 | (0.011, | 0.185) | |
| Trend^2^ ($\beta_{3}$) |  |  |  |  | |  | | -0.000 | 0.952 | (-0.001, | 0.001) | |
| Post-GST change in Trend^2^ ($\beta_{4}$) |  |  |  |  | |  | | -0.008 | 0.051 | (-0.015, | 0.000) | |
| CPI | 0.015 | 0.249 | (-0.012, | 0.041) | |  | | 0.012 | 0.429 | (-0.020, | 0.045) | |
| State-wise NDP | -0.400 | 0.764 | (-3.220, | 2.420) | |  | | -0.514 | 0.657 | (-2.953, | 1.925) | |
| R-squared |  | 0.095 |  |  | |  | |  | 0.103 |  |  | |

Note: The dependent variable is the year-on-year growth rate in state level purchases of aerated drinks. Both models are estimated with month and state fixed effects to account for state heterogeneity and seasonality. ^logarithm of state-wise net domestic product at factor cost at constant price (in Indian rupee). This data is obtained from the Reserve Bank of India to account for possible income effect on aerated drink purchases. It should be noted that unlike the CPI the NDP data does not distinguish urban and rural sectors and hence may be a noisy measure of income changes across urban Indian states. To our best knowledge, there is no available urban-specific monthly data on state-level GDP.

1. **Additional figures**

Figure D1. Monthly total purchases of aerated drinks (thousand litre) (in blue)


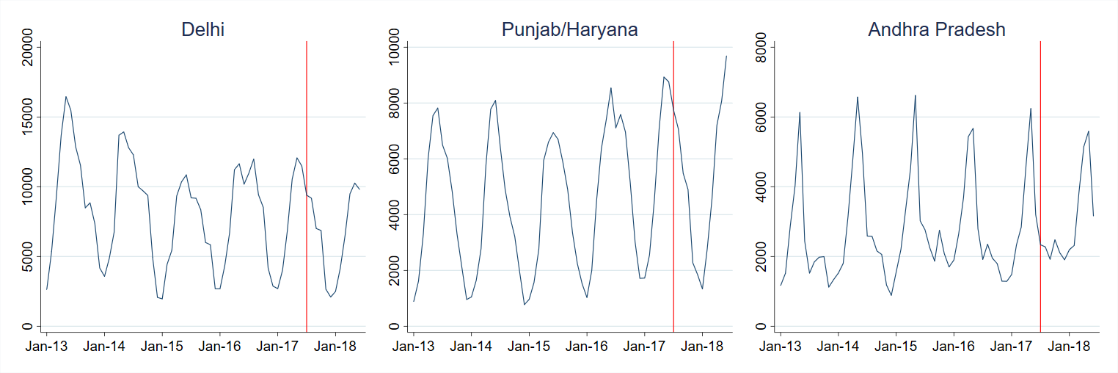

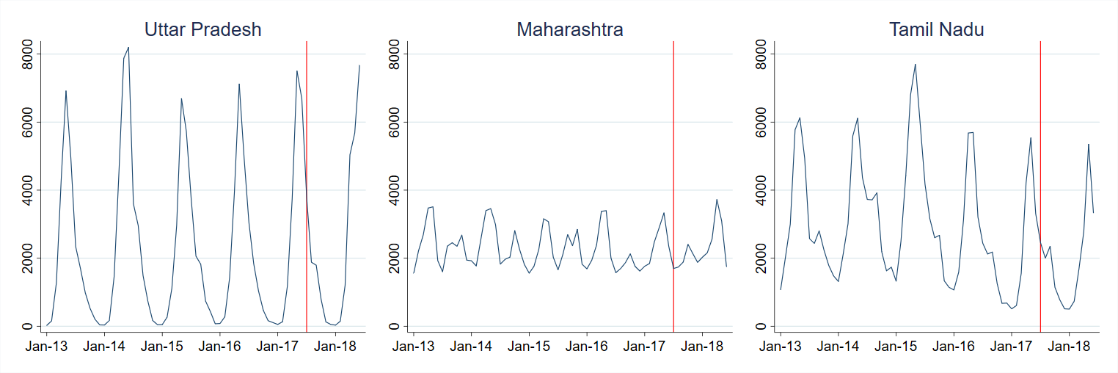

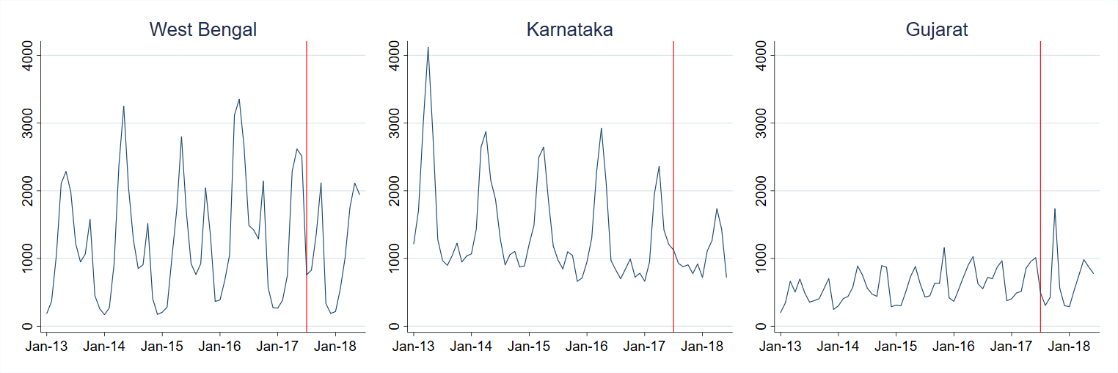

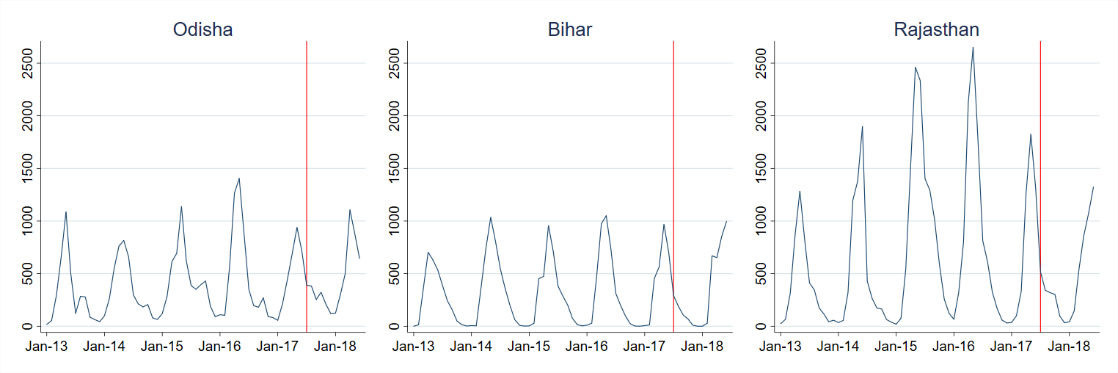

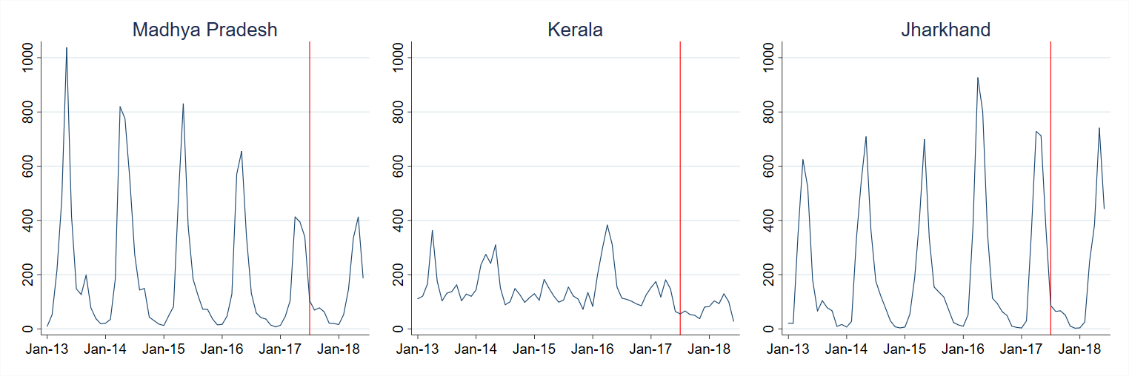


Note: The red vertical line indicates the implementation of GST in July 2017

Figure D2. State-level fitted values of year-on-year growth rate from model 2 (%) (in black)


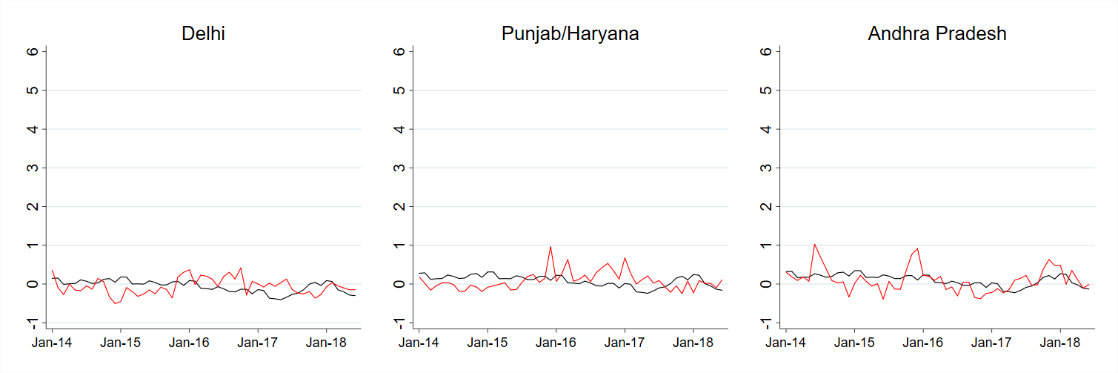

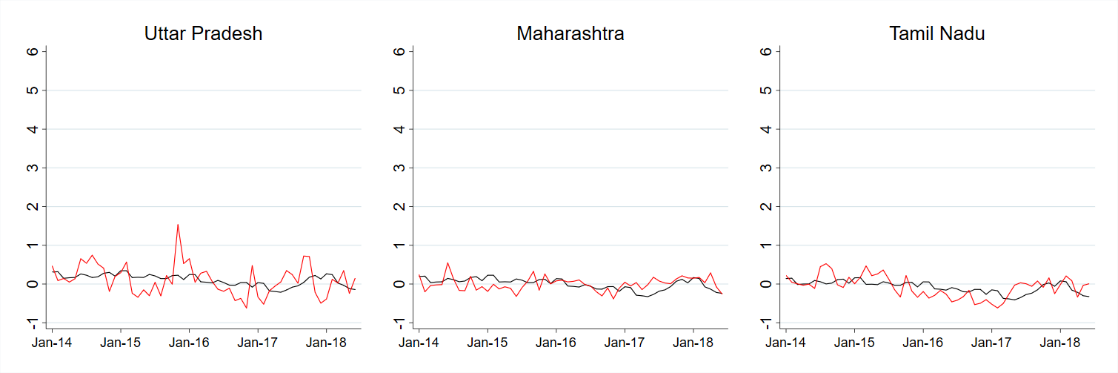

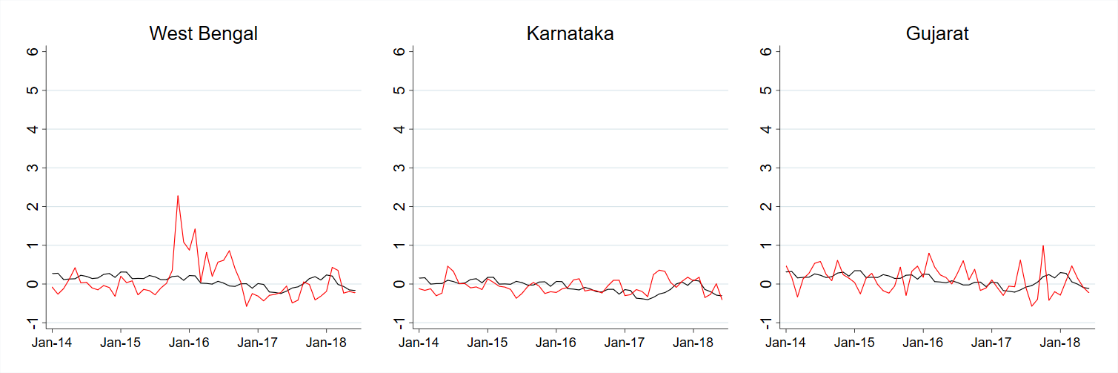

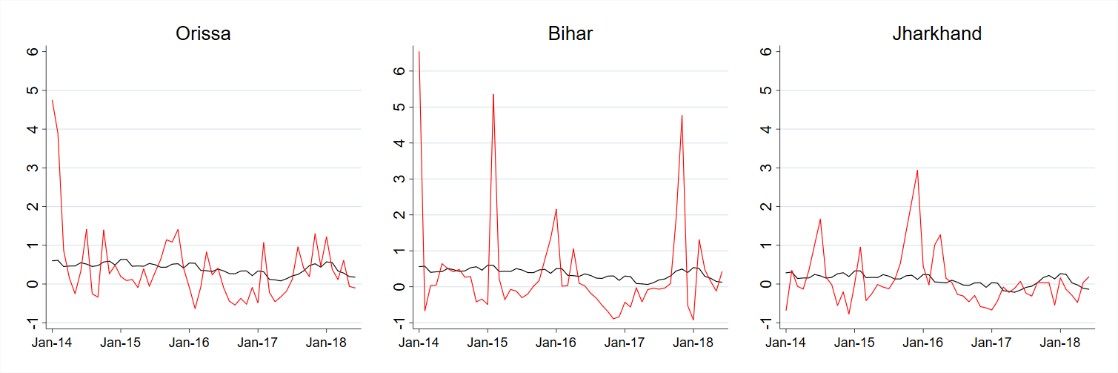


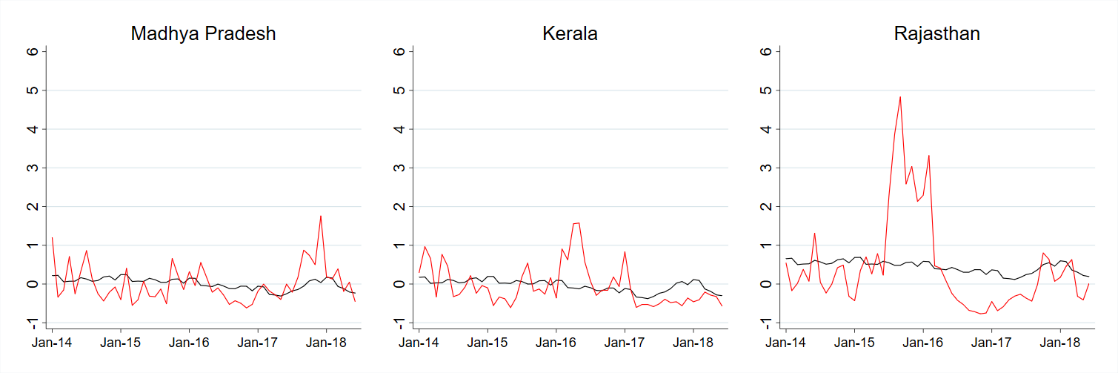


Note: The red line represents the actual values of year-over-year growth rate for each state. Observations from Rajasthan were excluded in the model estimation. For models 1,the graphs on fitted values are visually similar to the ones reported here with large residuals observed in West Bangel, Orissa, Bihar and Jharkhand.

**E. Stratified analysis**

Table E1: ITS estimates of post-GST changes for higher income urban states* (N=378)

|  | **Model 1: Linear trend change** | | | |  | **Model 2: Non-linear trend change** | | | |
| --- | --- | --- | --- | --- | --- | --- | --- | --- | --- |
|  | Coefficient | p-value | 95% CI | |  | Coefficient | p-value | 95% CI | |
| Trend ($\beta_{1}$) | 0.002 | 0.825 | (-0.017, | 0.021) |  | -0.041 | 0.530 | (-0.019, | 0.011) |
| Post-GST change in Trend ($\beta_{2}$) | -0.001 | 0.960 | (-0.034, | 0.033) |  | 0.037 | 0.521 | (-0.096, | 0.171) |
| Trend^2^ ($\beta_{3}$) |  |  |  |  |  | -0.000 | 0.205 | (-0.001, | 0.000) |
| Post-GST change in Trend^2^ ($\beta_{4}$) |  |  |  |  |  | -0.002 | 0.593 | (-0.012, | 0.008) |
| CPI | -0.012 | 0.504 | (-0.053, | 0.029) |  | 0.013 | 0.464 | (-0.018, | 0.044) |
| Constant | 1.503 | 0.525 | (-3.952, | 6.957) |  | 2.054 | 0.452 | (-4.198, | 8.306) |
| R-squared |  | 0.091 |  |  |  |  | 0.096 |  |  |

Note: *states with percentage of urban population above poverty line in 2011/12<10.5%, including Kerala, Delhi, Tamil Nadu, Maharashtra, Andhra Pradesh, Gujarat, Punjab/Haryana. The dependent variable is the year-on-year growth rate in state level purchases of aerated drinks. Both models are estimated with month and state fixed effects to account for state heterogeneity and seasonality.

Table E2: ITS estimates of post-GST changes for lower income urban states* (N=378)

|  | **Model 1: Linear trend change** | | | |  | | **Model 2: Non-linear trend change** | | | | |  |
| --- | --- | --- | --- | --- | --- | --- | --- | --- | --- | --- | --- | --- |
|  | Coefficient | p-value | 95% CI | | |  | | Coefficient | p-value | 95% CI | | |
| Trend ($\beta_{1}$) | -0.022 | 0.010 | (-0.036, | -0.007) | |  | | -0.022 | 0.207 | (-0.060, | 0.016) | |
| Post-GST change in Trend ($\beta_{2}$) | 0.035 | 0.170 | (-0.020, | 0.092) | |  | | 0.170 | 0.028 | (0.026, | 0.316) | |
| Trend^2^ ($\beta_{3}$) |  |  |  |  | |  | | -0.000 | 0.941 | (-0.001, | 0.002) | |
| Post-GST change in Trend^2^ ($\beta_{4}$) |  |  |  |  | |  | | -0.014 | 0.042 | (-0.026, | -0.001) | |
| CPI | 0.026 | 0.185 | (-0.017, | 0.069) | |  | | 0.024 | 0.464 | (-0.042, | 0.091) | |
| Constant | -3.291 | 0.196 | (-8.796, | 2.214) | |  | | -3.177 | 0.385 | (-11.474, | 5.120) | |
| R-squared |  | 0.109 |  |  | |  | |  | 0.124 |  |  | |

Note: *states with percentage of urban population above poverty line in 2011/12>10.5%, including West Bengal, Karnataka, Orissa, Madhya Pradesh, Jharkhand, Uttar Pradesh, Bihar. The dependent variable is the year-on-year growth rate in state level purchases of aerated drinks. Both models are estimated with month and state fixed effects to account for state heterogeneity and seasonality.
